# The Delineation of Management Zones of the *Halyomorpha halys* (Hemiptera: Pentatomidae) Population Based on Its Spatiotemporal Distribution for Precision Agriculture Purposes

**DOI:** 10.3390/insects16040336

**Published:** 2025-03-22

**Authors:** Vasileios Liakos, Eleni I. Koutsogeorgiou, Sofia Charouli, Ioannis E. Navrozidis, Georgios Proias, Stefanos S. Andreadis

**Affiliations:** 1 Laboratory of Precision Agriculture, Department of Agrotechnology, University of Thessaly, Gaiopolis, 41110 Larissa, Greece; sofiaharouli@yahoo.gr (S.C.); giproias@uth.gr (G.P.); 2 Laboratory of Applied Zoology and Parasitology, School of Agriculture, Aristotle University of Thessaloniki, 54124 Thessaloniki, Greece; eikoutso@agro.auth.gr; 3 Institute of Plant Breeding and Genetic Resources, Directorate General of Agricultural Research, Hellenic Agricultural Organization “Dimitra”, 57001 Thermi, Greece; sandreadis@elgo.gr; 4 Laboratory of Remote Sensing, Spectroscopy and GIS, School of Agriculture, Faculty of Agriculture, Forestry and Natural Environment, Aristotle University of Thessaloniki, 54124 Thessaloniki, Greece; navrozidisie@gmail.com

**Keywords:** brown marmorated stink bug, kiwifruit, variable rate application, pesticide, site-specific management, remote sensing

## Abstract

Precision Agriculture is an agricultural management strategy that aims to increase farmers’ profit, maximize crop productivity and sustainability, and protect the environment by applying inputs in optimum rates based on plant needs. The delineation of site-specific management zones is a crucial step at the application of Precision Agriculture. However, the procedure of delineating management zones for pest management is difficult since pest populations are dynamic and change spatially and temporally throughout a growing season. The objectives of this work is to study kiwi canopy characteristics, to correlate them with *Halyomorpha halys* (Hemiptera: Pentatomidae) populations and delineate management zones for pesticide applications in variable rates. To achieve this, four kiwi orchards in total were selected in the regions of Pieria and Imathia in Greece. Τen traps were installed from early May to late October within each selected kiwi orchard: two types of traps at every side of the orchards and the center. The installed traps were examined weekly, and the number of the captured *H. halys* was recorded. During the same days, sentinel satellite images were analyzed to calculate the indices: NDVI (Normalized Difference Vegetation Index) and NDWI (Normalized Difference Water Index). The collected data were combined in a GIS software to delineate management zones using a K means algorithm and unsupervised classification. The results of this three-year study showed population variability within the kiwi orchards since the population of *H. halys* was higher in field regions where NDVI and NDWI values were high. The delineation of management zones revealed that there are spatio-temporal stable zones in each field where there is high, medium, and low risk to develop *H. halys* populations. The benefits of the proposed strategy are multiple since it is expected that farmers will be able to reduce the production expenses of kiwifruits and environmental protection while increasing profit.

## Simple Summary

This study explores the use of Precision Agriculture techniques to manage the brown marmorated stink bug (*Halyomorpha halys*) in kiwi orchards, focusing on delineating management zones (MZs) for variable-rate pesticide applications by integrating pest monitoring data with canopy characteristics assessed through remote sensing indices. Pheromone traps were installed in four kiwi orchards in Greece over three growing seasons to monitor *H. halys* populations, and satellite-derived Normalized Difference Vegetation Index (NDVI) and Normalized Difference Water Index (NDWI) values were calculated to assess canopy health and moisture. A significant correlation was observed between *H. halys* population densities and areas with higher NDVI and NDWI values, indicating the pest’s preference for healthier, more humid canopy conditions. Using GIS-based K means clustering, this study delineated three risk-based MZs (low, medium, high) for each orchard and season, revealing stable spatial patterns over time. This site-specific approach allows for targeted pesticide applications, reducing chemical usage, minimizing environmental impacts, and lowering production costs.

## 1. Introduction

The primary goal of implementing new technologies in agriculture is to increase farmers’ production. Since 1960, a substantial decrease in crop losses due to pests has been observed [1]. However, roughly 45% of the global food produced annually is lost due to infestation by pests [2]. Furthermore, two million tonnes per year of pesticides are used worldwide, out of which 29.5% are insecticides. In Europe, despite the great amount of insecticide use, which accounts for nearly 12.5% of the total annual production costs, farmers persist in utilizing insecticides at the same levels. However, insecticides pose a significant threat to both human health and the environment. The use of chemical insecticides can cause harm to the skin, nervous system, reproductive organs, the endocrine system, and the digestive system [3]. Additionally, insecticides have the potential to contaminate groundwater and soil through the process of leaching [4].

The strategy of Precision Agriculture involves managing fields by taking into account the varying characteristics within fields, crops, and animals, through the application of several different methodologies and technologies. The primary objective of Precision Agriculture is to boost crop yields, protect the environment, and most importantly, reduce production expenses by utilizing optimized quantities of inputs that are tailored to the actual needs of the plants. This can lead to a significant decrease in pesticide use [5]. To accomplish these objectives, it is suggested to conduct a delineation of management zones (MZs) within fields, which are sub-regions in which specific combinations of factors that limit yield are uniformly distributed across the area [6,7]. MZs for insecticide applications could be mapped using a variety of data, depending on the application type. Due to the high dynamism of pest populations in space and time [8], data should be recorded at a high resolution. Additionally, zoning for pest management needs to take into account temporally dynamic factors [9]. Furthermore, clustering algorithms, which are used to delineate MZs, may not effectively identify meaningful management zones [10]. The mapping of various agricultural zoning factors is accomplished through the application of image analysis to derive specific metrics (vegetation metrics, soil metrics, etc.) and geostatistical interpolation techniques such as kriging [11,12,13,14,15]. The optimal number of zones is a crucial element in the effectiveness of Multiple Zoning Systems. Collected data are subjected to statistical tests including spatial correlation analysis, principal component analysis, and spatially weighted principal component analysis to determine the most suitable number of zones in each field.

The brown marmorated stink bug, *Halyomorpha halys* (Stål) (Hemiptera: Pentatomidae), is a polyphagous pest that was unintentionally introduced into both the United States and Europe from East Asia [16,17,18]. *Halyomorpha halys* quickly accumulates large populations on a wide range of host plants both within and near orchards and crop fields, resulting in substantial economic losses for farmers [19,20]. The existence of *H. halys* led to significant damage in apple, peach, and pear orchards [16,21]. Since its initial discovery in Liechtenstein in 2004 [22], *H. halys* has become a primary key pest in numerous European fruit orchards [23,24,25,26,27], vegetable crops [28], kiwifruit [29], and hazelnuts [30] over the past few years. Furthermore, *H. halys* is viewed as a significant nuisance pest for residents primarily because adult insects that survive the winter months migrate to and establish themselves within residential buildings and other artificial structures in considerable quantities during autumn [18,31].

*Halyomorpha halys* is considered a threat originating from a field’s perimeter; thus, numerous researchers concentrate on creating new methods for insecticide application at crop field boundaries rather than across fields [32,33,34,35,36]. Research studies typically rely on data collected from pheromone traps positioned solely at the perimeters of crop fields, as opposed to throughout the fields, for tracking *H. halys* populations [37,38,39].

The spatio-temporal study and precise recognition of kiwi canopy characteristics might be essential to detect canopy damage inflicted by *H. halys* and the resulting economic losses. The early identification of *H. halys* infestations in kiwifruit orchards may be enhanced using multispectral remote sensing that can offer insights into pest conditions in agricultural fields and has been employed to detect pest-induced crop damage in sorghum [40], cotton, and wheat [41,42,43,44,45]. The Normalized Difference Vegetation Index (NDVI) is an essential parameter for assessing the vigor of vegetation and detecting plant damage. The NDVI defines values from −1 to 1, where negative values are mainly associated with clouds, water, and snow, while values close to zero are indicative of bare soil. Moderate NDVI values signify regions lacking vegetation, whereas large NDVI values (close to 1) reflect healthy vegetation/high canopy density. A range of NDVI information obtained from remote sensing imagery has led to NDVI time series from remote sensing data [46,47]. Nonetheless, there is insufficient research connecting the NDVI to the presence of insects. Willers et al. [48] identified a spatial relationship between the tarnished plant bug *Lygus lineolaris* (Palisot de Beauvois) (Hemiptera: Miridae) and areas of high cotton growth (NDVI). The Normalized Difference Water Index (NDWI) is another critical parameter that has a significant relationship with water content in plant leaves. The NDWI can range from −1 and 1 and responds more quickly than the NDVI to changes in water availability. As leaf water content decreases, NDWI values also decrease and reverse. This index has been utilized to investigate the remote sensing potential for mapping and monitoring vegetation water content in corn and soybean canopies [49], to characterize land cover and vegetation type [50,51] and, last but not least, to monitor water stress in semiarid regions [52]. As with the NDVI, there are few studies on the use of the NDWI and pest control. McFeeters et al. [53] used the NDWI in conjunction with geographic information systems to detect swimming pools to reduce mosquito populations. Jokar et al. [54] studied the distribution of the cotton bollworm, *Helicoverpa armigera* (Hübner) (Lepidoptera: Noctuidae), and created inundation maps using NDWI data from the Sentinel 1 satellite. Finally, Prabhakar et al. [55] successfully detected invasive fall armyworms by extracting NDWI data from Sentinel 2A satellite imagery.

In commercial fields, broad-spectrum insecticides are the primary tool used to control *H. halys* populations. However, pollinators and natural enemies are frequently poisoned by the herbicides that work well against *H. halys*. Farmers and other interested parties are therefore looking for innovative ways to lessen the usage of pesticides to manage *H. halys* populations.

In this work, the collected NDVI and NDWI data from Sentinel-2 imagery are presented along with *H. halys* population data gathered using pheromone traps during three growing seasons in Greece. Both temporal and spatial analyses were performed on these data. The goal of this research is to delineate MZs that are useful in predicting *H. halys* populations spatially and temporally. Farmers will use the results to apply insecticides at variable rates rather than the usual uniform rates in order to reduce the amount of insecticides applied, increase their profits, and protect the environment and other beneficial insects. The European Union subsidizes the use of variable rate applications of insecticides through Precision Agriculture funding programs. The objectives of this study are to (a) understand the spatial development of the *H. halys* population within kiwi orchards in three different seasons and (b) to investigate if there are spatially and temporally similar MZs with kiwi orchards for insecticide applications.

## 2. Materials and Methods

### 2.1. Field Sites and Traps

During the 2021, 2022, and 2023 growing seasons, a field survey was conducted on four kiwifruit orchards located at Dion, Nea Efessos, Episkopi, and Meliki in the prefectures of Imathia and Pieria, in Greece. Table 1 shows the field characteristics of each kiwifruit orchard. All kiwifruit orchards were uniformly irrigated and fertilized. Farmers treated kiwifruit orchards without insecticides against *H. halys*, which provided an opportunity to detect seasonal changes in *H. halys* at several locations. The kiwi variety “Haywarth” was cultivated in all kiwifruit orchards, and the soil type of each experimental site was categorized as “silt”.

**Figure 1:**
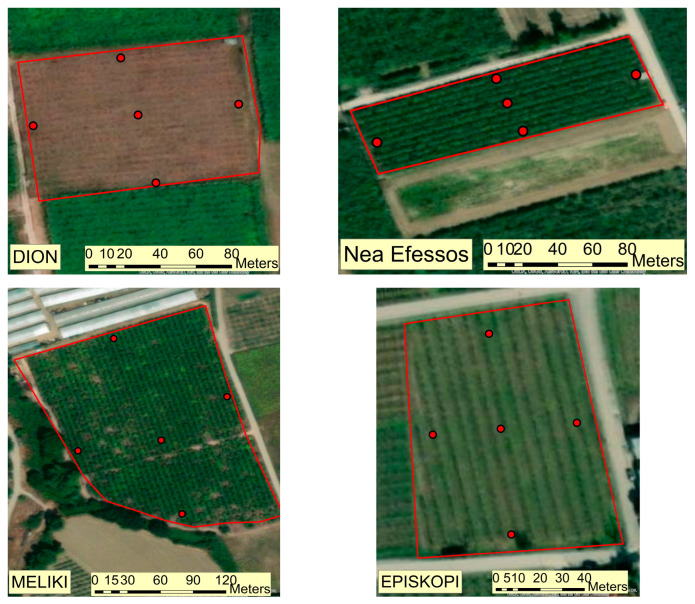
Kiwi orchards at different regions in Greece. Every orchard was equipped with two different types of traps at five sites. Red dots represent the exact positions where traps were installed.

Two trap types used for monitoring *H. halys*: (a) Rescue^®^ hanging stink bug rocket trap (Serbios S.r.l., Badia Polesine, Italy) and (b) sticky panel trap (Trécé Inc., Adair, OK, USA). Both trap types were installed in the tree canopy and baited with the available standard lures commercially produced by Serbios S.r.l. or Trécé Inc, respectively. The traps were inspected weekly, and the number of captured adult and nymphs of *H. halys* was recorded from early May to late October from 2021 to 2023. The installation strategy of traps was the same as described by Liakos et al. (2023) [15]. Figure 1 represents the sites where both types of traps were installed in each field.

### 2.2. Plant Canopy Characteristics

Sentinel satellite images with temporal resolution of five days were analyzed to calculate the Normalized Difference Vegetation Index (NDVI) [56] as well as the Normalized Difference Water Index (NDWI) [53]. NDVI values provide information about the vigor and the density of a canopy. NDVI values close to 1 indicate that the canopy is vigorous, while NDVI values close to or below 0.2 indicate deficiencies in the canopy. On the other hand, the NDWI value indicates the moisture content of the canopy. The higher the NDWI value, the higher the moisture content. After acquiring satellite images from May to October of each year and at the end of each growing season, NDVI and NDWI maps were created by averaging the pixel values of NDVI and NDWI values from all satellite images for each season (early, mid, and late season). Spatial analysis and mapping of average NDVI and NDWI values were performed using ArcGIS (Version 10.8, ESRI, Redlands, CA, USA). The final NDVI and NDWI maps for each season were generated using the Map Algebra (raster calculation) tool of ArcGIS.

### 2.3. Delineation of Management Zones

The collected spatio-temporal NDVI and NDWI data were combined together to delineate management zones since both indices are positively related to the *H. halys* populations—adults and nymphs [15]. The ArcGIS tool grouping analysis and especially the K Means algorithm were used to delineate the zones. This tool utilizes unsupervised machine learning methods to group data in a dataset. Unsupervised classification is known as a user-friendly, easy method since it does not require a set of pre-classified features to train the classification model to determine the groupings of the data [57].

The goal of the K Means algorithm is to partition features so the differences among the features in a group, over all groups, are minimized. The K Means algorithm works by first identifying seed features used to grow each group. Consequently, the number of seeds will always match the number of groups. The first seed was selected randomly. Selection of remaining seeds, however, while still employing a random component, applied a weighting that favors selection of subsequent seeds farthest in data space from the existing set of seed. Once the seed features were identified, all features were assigned to the closest seed feature. For each cluster of features, a mean data center was computed, and each feature was reassigned to the closest center. The process of computing a mean data center for each group and then reassigning features to the closest center continues until group membership stabilizes [57].

Another advantage of the grouping analysis tool is that it calculates and recommends the ideal number of zones that should be delineated for each dataset [57]. The recommended number of zones for the datasets of this work is three.

### 2.4. Statistical Analysis

Normally distributed data were analyzed using Pearson’s correlation (r) of the captured adult and nymph *H. halys* in IBM SPSS Statistics (Version 29). Statistical analysis focused on the correlation between NDVI, NDWI, and population densities of *H. halys* using the linear regression tool of the software. Each growing season was split up into three sampling periods (Table 2) with roughly equal trapping intervals to improve statistical analysis and understand the temporal preferences of *H. halys* [24,33,34,37,38,39,58]. The Cohen’s kappa index [59] was calculated using the IBM SPSS Statistics (Version 29) to measure the spatial consistency of the management zones through the seasons and years. Cohen’s kappa index close to zero means that the agreement between management zones is no better than chance, while Cohen’s kappa index close to one indicates a perfect agreement.

## 3. Results

### 3.1. Population Densities and Remotely Sensed Indices

**Figure 2:**
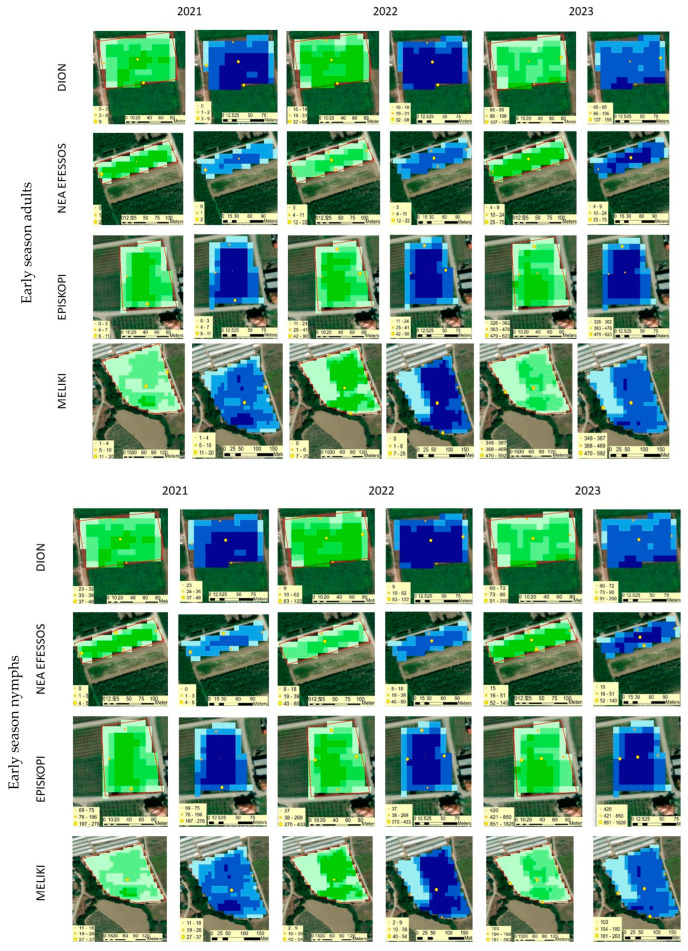

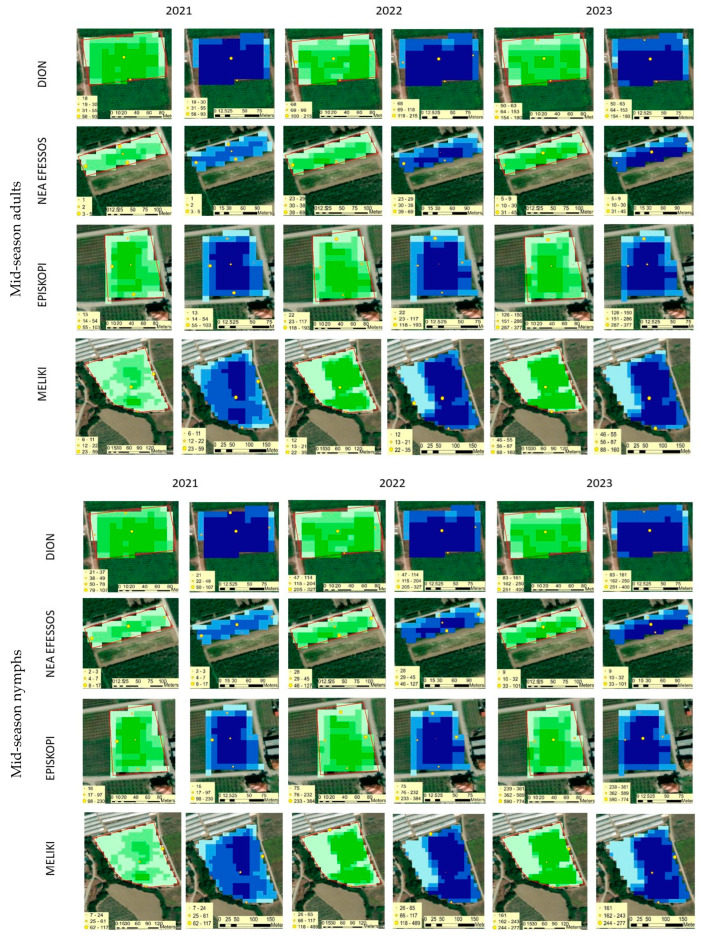

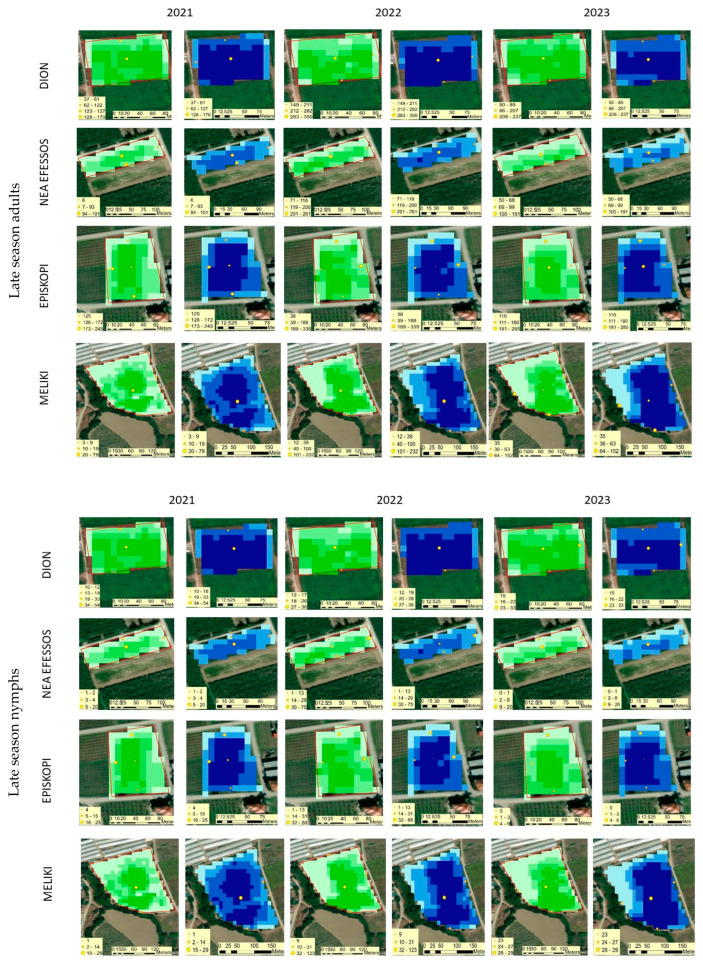
NDVI, NDWI, and *H. halys* spatial variability over three years of study for each season and fields based on adult and nymph captures. Green maps represent the NDVI variability. The brighter the green, the higher the NDVI values. The lowest NDVI is 0.62, and the highest is 0.95. Blue maps represent the NDWI variability. The darker the blue, the higher the moisture at the canopy and the opposite. The lowest NDWI value is −0.2, and the highest is 0.1. The yellow circles with red outlines show the exact site of the traps installed. The size of the yellow circles represents the size of the *H. halys* population. The bigger the circle, the bigger the population. The classification method for NDVI and NDWI data is the quantile.

Mapping and analyzing the variability of indices such as the NDVI and NDWI led to the understanding of *H. halys* adults and nymph variability in each field during three different seasons—early, mid-, and late (Figure 1). The annual seasonal NDVI and NDWI maps at Figure 2 were created by calculating the mean values for each pixel for the time period assigned to each season. The classification method “quantile” was used to map the spatio-temporal variability of both indices. This method assigns the same number of data values to each class. The outcome of the visual comparison of the size of *H. halys* populations (sum of *H. halys* individuals caught by both type of traps) at different sites relative to the values of the indices at those specific sites demonstrates that *H. halys* tends to develop populations at sites where the plant canopy is healthy (high NDVI values) and moisture is present in the canopy (high NDWI values).

The seasonal statistical analysis of *H. halys* populations, along with the NDVI and NDWI, showed that there was a significant correlation between them in the three survey years (Table 3), except for 2021, and that there is no significant correlation between *H. halys* density and the NDWI. This may have happened because, during the early season of each year and especially in 2021, the weather was often cloudy, and, therefore, we acquired less images than the other seasons. This explains the stronger correlations among *H. halys* density, the NDVI, and the NDWI during the mid- and late seasons rather than the early season. Nevertheless, the results suggest that *H. halys* tends to develop populations at places where the plant canopy is healthy (high NDVI values), and moisture is present in the canopy (high NDWI values).

### 3.2. Management Zones

**Figure 3:**
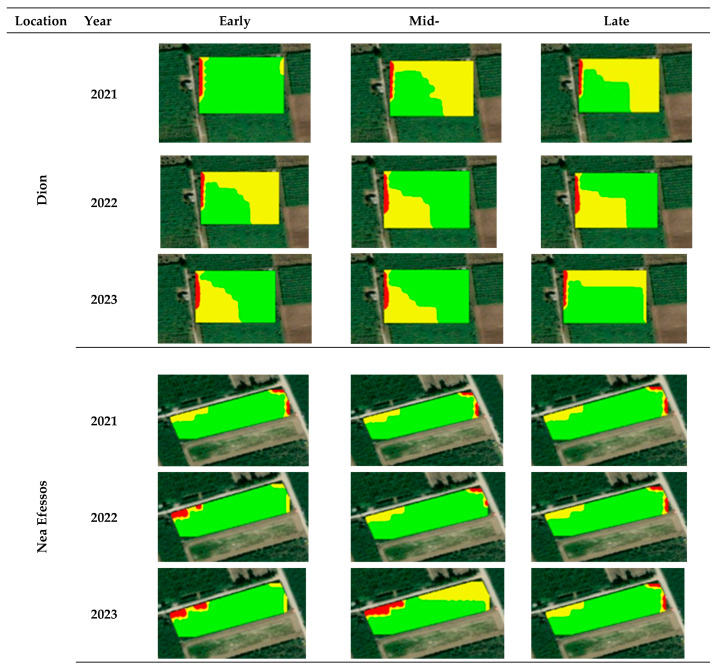

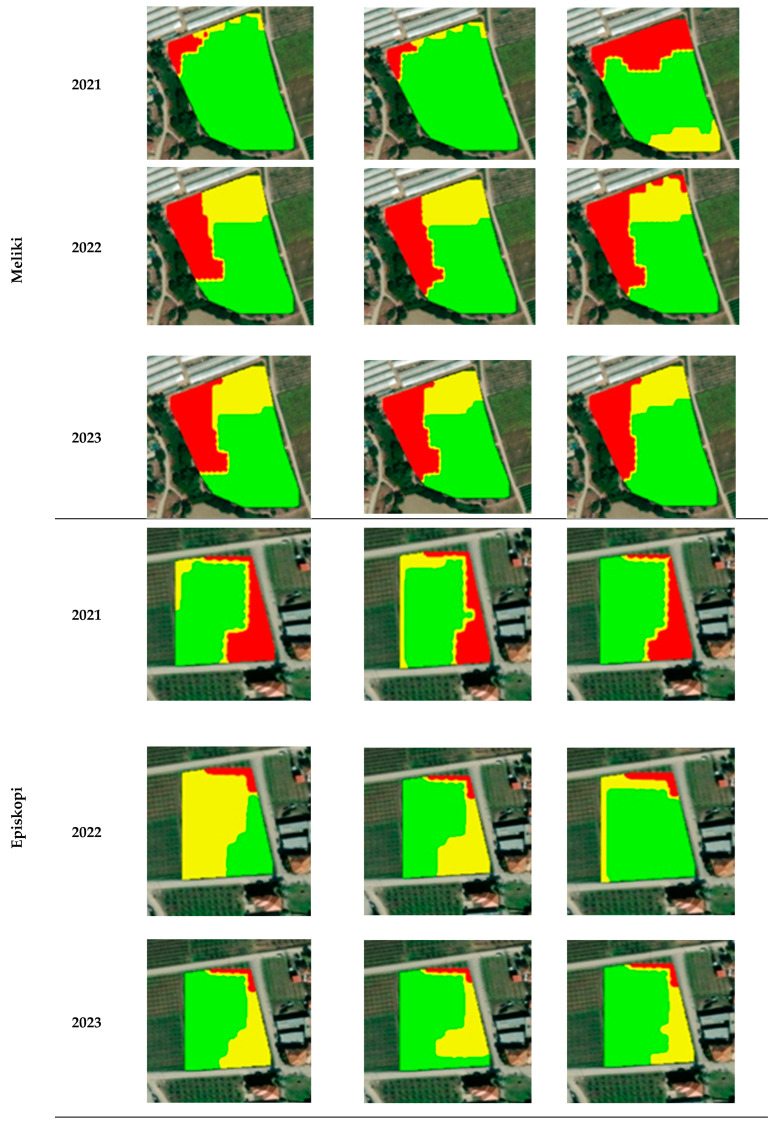
Spatial temporal management zones in each season for each field separately. Areas with green color are high-risk areas where *H. halys* could develop populations. On the other hand, the yellow color represents areas where there is medium risk to develop *H. halys* populations, and the red color shows the low-risk areas.

The delineation of management zones for pesticide applications revealed that, geostatistically, each site could be divided into three zones—low, medium and high risk—to develop *H. halys* populations. Figure 3 presents the annual management zones for each experimental site for each season (early, mid, and late). It is obvious that there are similar spatio-temporal zones through the different seasons in every field. The zones at Dion have the same shape and almost the same size in every year except for the early stage of 2021 and late stage of 2023. At Nea Efessos, the spatial pattern of the zones is the same, but the risk level of the zones changes between medium risk and low risk through the years. At Meliki field, the zones in 2021 are spatially different than the zones in 2022 and 2023, where the zones are almost spatially the same in both years. At Episkopi field, the zones had almost the same size and shape through the years except for the zone in 2022 at the late season.

The size of the zones is very important information for farmers because the pesticide applications will be based on the zones. Thus, Table 4 presents the size of the annual zones for each site and season as well as the average percent of each zone coverage compared to the total area of the field. The high-risk areas cover the highest percent of each field in every season and year. The percent of the low-risk areas varies from 3.6% (Nea Efessos, late season 2023) to 22.9% (Meliki, late season 2023). On the other hand, the percent of medium-risk areas varies from 7.1% (Nea Efessos, early season 2021) to 40.1% (Dion, med-season 2022). It is worth mentioning that the size of the percent of coverage of the medium risk zones is always greater or equal to low-risk zones and lower than the high-risk zones.

The Cohen’s kappa values were calculated to measure the spatial consistency of the management zones. The results of their spatial consistency are presented in Table 5 and Table 6. According to Table 5, there is near perfect agreement of the management zones between mid- and late seasons in most of the years and locations except for Dion in 2023, Nea Efessos in 2023, and Episkopi in 2022 where there is substantial agreement. As far as the early–mid-seasons is concerned, it was noticed that there is near perfect agreement of the management zones in most of the sites except for Dion in 2021 (substantial agreement) and 2022 (moderate agreement), Nea Efessos in 2023 (substantial agreement), and Episkopi in 2022 (substantial agreement). Similar results were recorded in management zones between the early–late season except for Dion in 2023 (substantial agreement), Meliki in 2021 (substantial agreement), and Episkopi in 2022 (moderate agreement).

The comparison of the management zones (Table 6) among the years at Dion revealed substantial agreement, except for the mid-season (near perfect agreement), and late season between 2022 and 2023 (moderate agreement). At Nea Efessos, our results indicate near perfect agreement at the management zones, except for the early season between 2021 and 2022, and 2021 and 2023, the mid-season between 2021 and 2023, and 2022 and 2023. Similar results were recorded in Meliki, but there is moderate agreement in the late season between 2021 and 2023. Finally, at Episkopi, there is fair agreement in the early season between 2021 and 2022, and 2022 and 2023. In the mid-season of 2022 and 2023, there is near perfect agreement, in the late season of 2022 and 2023, there is substantial agreement, while, in the rest of the cases, there is moderate agreement.

## 4. Discussion

There are few studies on *H. halys* in kiwifruit orchards using satellite imagery to extract information on vegetation characteristics such as the NDVI and NDWI. Zhu et al. [60] developed a model based on the NDVI and other data to predict the spatial distribution of *H. halys* at a large scale. Reisig et al. [61] used NDVI data to estimate the damage already caused by stink bugs to cotton plants. Liakos et al. [15] utilized NDVI and NDWI data to predict *H. halys* populations in a two-year study. The NDVI provides information on kiwi canopy vigor and density. Higher NDVI values mean that the canopy is greener and has a high density of leaves, creating shade under the plants. This is consistent with the results of other studies that concluded that *H. halys* prefers dark areas over light areas [15,62]. Moreover, in the current study, more *H. halys* were trapped in the areas where the NDWI was high, i.e., where canopy moisture was high. This is in contrast to Cullum et al. [63], who reported that *H. halys* preferred dry rather than moist areas. The difference in the results may be explained by the fact that Cullum et al. [63] conducted the experiment in shelters and not in an orchard, where the microclimate changes rapidly based on canopy and environmental factors. In that scope, the NDVI and NDWI are considered very important indicators for *H. halys* population development in our study. Ambient temperature is known to play an important role in the development of *H. halys* populations [18,64,65], but it is difficult to map the temperature variability within a kiwi orchard because of the microclimate changes. The NDVI that shows the sites with a high density of leaves and shade could be used to indirectly understand the temperature variability under the kiwi canopy. This could be studied in future work.

From the above, it is clear that the NDVI and NDWI play very important roles in the establishment of *H. halys* populations. Knowing the factors that affect *H. halys* populations, as well as the timing and site of *H. halys* presence, can help farmers to manage their orchards efficiently by applying agrochemicals or natural enemies at the right time and place according to Precision Agriculture practices, rather than uniformly as they usually do. These new Precision Agriculture practices typically reduce production costs and protect the environment.

The success of Precision Agriculture application relies on the delineation of correct spatio-temporal management zones. At this important step, every field is divided into specific areas where farmers could apply insecticides in variable rates based on the *H. halys* populations. This work presents a novel methodology of delineating useful site specific management zones to treat *H. halys* populations in kiwi orchards (in field scale). The variability of canopy characteristics such as the NDVI and NDWI was taken into consideration during the delineation of management zones since it has already proved that there is positive correlation between both indices and the presence of *H. halys* populations [15]. The combination of the collected data to export the management zones as well as the optimum number of zones for each dataset was performed in ArcGIS software. The data analysis as well as the delineation of management zones focused on three different seasons within each growing season because *H. halys* have different distributions within fields based on their biological processes. At this work, the spatio-temporal distribution within the experimental sites was great. This is in agreement with other work [66,67] that have mentioned the importance of taking into consideration temporally dynamic zone characteristics to treat pest populations. However, traps alone (by themselves) do not always accurately monitor the spatial distribution of a pest population due to several issues such as stage-specific limitations or unfavorable microhabitats [18]. Thus, visual scouting as well as remotely sensed data to delineate management zones could be considered complementary tools for monitoring *H. halys* population distribution. The zones should be categorized into classes of the level of risk of *H. halys* populations. In this work, the zones were classified into low, medium and high risks of *H. halys* presence. The high-risk zone could be translated to more than 10 adults per trap per week, the medium risk zone to 5–10 adults per trap per week, and the low risk zone to less than 5 adults per trap per week [34]. Consequently, it could be proposed to apply 100, 50, and 10% of the amount of pesticides that farmers usually use at the high, medium, and low-risk zones, respectively (more research should be conducted on the pesticide rates). However, the percentile size that the high-risk zones cover at the four experimental sites was the highest compared to the other two classes and varied from 54.1 to 87.5%. This means that kiwi producers can save at least 12.5% of the total insecticides by applying them in variable rates. The fact that the high-risk areas are dominant compared to the other two zones may be caused by different environmental conditions such as the microclimate, type of crops cultivated at the nearest fields, as well as canopy characteristics such as the high density of leaves and leaf moisture. Additionally, the shape and the size of the zones are ideal for the variable rate application of pesticides because the zones are distinct, and it is not required to change the rate frequently, which usually produces errors.

Up until now, there are no other studies about the delineation of management zones for *H. halys*. However, similar research but with a completely different approach was carried out by the authors of [58], who studied the spatio-temporal distribution of *H. armigera* and *Pectinophora gossypiella* (Saunders) (Lepidoptera: Gelechiidae) in a cotton field at a large scale and developed spatio–temporal zones. The developed zones were similar with each other. This is consistent with the results of this work, where it is clear that there are steady management zones among the different seasons and years. This was proven by the Cohen’s kappa index that was calculated for the delineated management zones for different seasons and years. The lowest recorded Cohen’s kappa index was 0.34, which means fair agreement between the management zones, and the highest was 0.99, which means near perfect agreement. The fact that there are stable spatio—temporal zones can help farmers understand the distribution of *H. halys* populations in each field. However, it should be mentioned that this work refers to the *H. halys* population. Further study is needed for the delineation of management zones for insect populations. The next step of this study is to develop a smart app for smartphones that will allow users to access NDVI and NDWI data. This app will be programmed to dynamically de-lineate management zones for variable rate applications of insecticides in kiwi orchards.

## Figures and Tables

**Table 1:** Coordinates in decimal degrees and orchard size in hectares for each kiwi orchard.

Prefecture	Location	Longitude	Latitude	Size (ha)
Pieria	Dion	22.491933	40.170259	0.69
	Nea Efessos	22.489333	40.221147	0.56
Imathia	Episkopi	22.127619	40.689933	0.70
	Meliki	22.404548	40.519695	2.36

**Table 2:** Months assigned for each seasonal period.

Months	Season		
	Early	Mid-	Late
May–June	X		
July–August		X	
September–October			X

**Table 3:** Seasonal correlation between *H. halys* population densities and indices extracted from satellite images.

				**Early Season**					
	**2021**			**2022**			**2023**		
	**Density**	**NDVI**	**NDWI**	**Density**	**NDVI**	**NDWI**	**Density**	**NDVI**	**NDWI**
Density	1			1			1		
NDVI	0.72 *	1		0.75 *	1		0.79 *	1	
NDWI	0.68	0.79 *	1	0.71 *	0.73 *	1	0.70 *	0.72 *	1
				**Mid-Season**					
	**2021**			**2022**			**2023**		
	**Density**	**NDVI**	**NDWI**	**Density**	**NDVI**	**NDWI**	**Density**	**NDVI**	**NDWI**
Density	1			1			1		
NDVI	0.75 *	1		0.80 **	1		0.72 *	1	
NDWI	0.81 **	0.75 *	1	0.79 **	0.78 *	1	0.72 *	0.77 *	1
				**Late Season**					
	**2021**			**2022**			**2023**		
	**Density**	**NDVI**	**NDWI**	**Density**	**NDVI**	**NDWI**	**Density**	**NDVI**	**NDWI**
Density	1			1			1		
NDVI	0.80 **	1		0.82 **	1		0.85 **	1	
NDWI	0.72 *	0.70 **	1	0.75 **	0.79 **	1	0.82 **	0.84 **	1

* correlation is significant at the 0.05 level. ** correlation is significant at the 0.01 level.

**Table 4:** Size of each zone in hectares and average percent of zonal coverage.

Field	Year	2021			2022			2023		
		Early			Mid-			Late		
		Low	Medium	High	Low	Medium	High	Low	Medium	High
Dion	2021	0.03	0.04	0.62	0.03	0.35	0.31	0.03	0.37	0.29
	2022	0.03	0.34	0.32	0.05	0.25	0.39	0.04	0.26	0.39
	2023	0.05	0.25	0.39	0.04	0.23	0.42	0.03	0.17	0.49
	Avg (%)	5.3	30.4	64.3	5.8	40.1	54.1	4.8	38.6	56.5
Nea Efessos	2021	0.02	0.05	0.49	0.02	0.05	0.49	0.02	0.05	0.49
	2022	0.03	0.03	0.50	0.01	0.04	0.51	0.02	0.05	0.49
	2023	0.04	0.04	0.48	0.06	0.15	0.35	0.02	0.05	0.49
	Avg (%)	5.4	7.1	87.5	5.4	14.3	80.4	3.6	8.9	87.5
Meliki	2021	0.10	0.07	2.19	0.08	0.07	2.21	0.61	0.13	1.62
	2022	0.47	0.50	1.39	0.48	0.50	1.38	0.50	0.35	1.51
	2023	0.47	0.50	1.39	0.51	0.49	1.36	0.51	0.49	1.36
	Avg (%)	14.7	15.1	70.2	15.1	15.0	69.9	22.9	13.7	63.4
Episkopi	2021	0.20	0.12	0.38	0.18	0.15	0.37	0.20	0.09	0.41
	2022	0.03	0.42	0.23	0.02	0.23	0.45	0.03	0.23	0.44
	2023	0.02	0.23	0.45	0.02	0.21	0.47	0.02	0.18	0.50
	Avg (%)	11.9	36.7	50.5	10.5	28.1	61.4	11.9	23.8	64.3

**Table 5:** Cohen’s kappa index of management zones between annual seasons.

Location	Year	Early–Mid	Mid–Late	Early–Late
Dion	2021	0.63	0.94	0.62
	2022	0.45	0.98	0.47
	2023	0.96	0.63	0.68
Nea Efessos	2021	0.99	0.99	0.99
	2022	0.88	0.94	0.96
	2023	0.74	0.63	0.71
Meliki	2021	0.98	0.68	0.69
	2022	0.98	0.85	0.87
	2023	0.97	0.98	0.98
Episkopi	2021	0.93	0.90	0.95
	2022	0.65	0.73	0.60
	2023	0.92	0.89	0.91

**Table 6:** Seasonal Cohen’s kappa index of management zones between years.

Location	Season	2021–2022	2022–2023	2021–2023
Dion	Early	0.62	0.65	0.63
	Mid	0.64	0.96	0.62
	Late	0.61	0.57	0.64
Nea Efessos	Early	0.79	0.96	0.78
	Mid	0.95	0.77	0.75
	Late	0.97	0.97	0.97
Meliki	Early	0.82	0.98	0.81
	Mid	0.71	0.96	0.72
	Late	0.61	0.94	0.60
Episkopi	Early	0.34	0.35	0.56
	Mid	0.57	0.89	0.59
	Late	0.58	0.62	0.59

## Data Availability

The original contributions presented in this study are included in the article. Further inquiries can be directed to the corresponding author.
